# External validation of EORTC risk scores to predict recurrence after transurethral resection of brazilian patients with non-muscle invasive bladder cancer stages Ta and T1

**DOI:** 10.1590/S1677-5538.IBJU.2015.0169

**Published:** 2016

**Authors:** Gilberto L. Almeida, Wilson F. S. Busato, Carmen Marcondes Ribas, Jurandir Marcondes Ribas-Filho, Ottavio De Cobelli

**Affiliations:** 1 Universidade do Vale do Itajaí, SC, Brasil/Instituto Catarinense de Urologia (INCAU), Itajaí, Brasil; Instituto Catarinense de Urologia, Itajaí, Brasil; 2 Faculdade Evangélica do Paraná (FEPAR)/Instituto de Pesquisas Médicas (IPEM), Curitiba, Brasil; 3 Università degli Studi di Milano, Milano, Italia; 4 Dipartimento di Urologia, Istituto Europeo di Oncologia (IEO), Milano, Italia

**Keywords:** Urinary Bladder, Carcinoma, Recurrence, Transurethral Resection of Prostate

## Abstract

Validate the EORTC risk tables in Brazilian patients with NMIBC.

**Methods:**

205 patients were analyzed. The 6 parameters analyzed were: histologic grading, pathologic stage, size and number of tumors, previous recurrence rate and concomitant CIS. The time for first recurrence (TFR), risk score and probability of recurrence were calculated and compared to the probabilities obtained from EORTC risk tables. C-index was calculated and accuracy of EORTC tables was analyzed. Results: pTa was presented in 91 (44.4%) patients and pT1 in 114 (55.6%). Ninety-seven (47.3%) patients had solitary tumor, and 108 (52.7%) multiple tumors. One hundred and three (50.2%) patients had tumors smaller than 3 cm and 102 (40.8%) had bigger than 3 cm. Concomitant CIS was observed in 21 (10.2%) patients. Low grade was presented in 95 (46.3%) patients, and high grade in 110 (53.7%). Intravesical therapy was utilized in 105 (56.1%) patients. Recurrence was observed in 117 (57.1%) patients and the mean TFR was 14,2 ± 7,3 months. C-index was 0,72 for 1 year and 0,7 for 5 years. The recurrence risk was 28,8% in 1 year and 57,1% in 5 years, independently of the scoring risk. In our population, the EORTC risk tables overestimated the risk of recurrence in 1 year and underestimated in 5 years.

**Conclusion:**

The validation of the EORTC risk tables in Brazilian patients with NMIBC was satisfactory and should be stimulated to predict recurrence, although these may overestimated the risk of recurrence in 1 year and underestimated in 5 years.

## INTRODUCTION

At least 85% of patients with bladder cancer (BCa) present the disease confined to the mucosa (Ta and Tis) or submucosa (T1), representing non-muscle invasive bladder cancer (NMIBC). These are heterogeneous tumors with high variation of reported recurrence rates in literature. They vary from 15–61% at first year to 31–78% at five years (1, 2). Stratification is responsible for the correct choice of treatment, based on recurrence and progression risks (3).

European Organization for Research and Treatment of Cancer (EORTC) has published a score system based on the risks of progression and recurrence of patients with NMIBC following TRUS (4). Recently, the European Association of Urology (EAU) has adopted in its guidelines the risk tables and recommends stratification of NMIBC with low, intermediate and high risk of recurrence (5). However, external validations are necessary to adopt these risk tables in clinical practice and in different populations (6–9).

Although score EORTC risks are widely used in daily urological practice, there are not enough data from South America, particularly from Brazil, to apply them in the Brazilian population.

## MATERIAL AND METHODS

Prospectively and consecutively, 205 patients with NMIBC stages Ta and T1 submitted to bladder TRUS resection, from January 2003 to October 2010 were analyzed. Patients were treated at the Department of Uro-oncology of Instituto Catarinense de Urologia in Itajaí, Santa Catarina, Brazil. The patients were attended by the public health service and private practice and had all socio-economic status. Ethnic background was not considered, since it is virtually impossible to distinguish these characteristics in this widely genetic and ethnic variant population.

New and recurrent cases were considered. It was excluded patients submitted to previous radiotherapy and chemotherapy due to other tumors throughout the period of study, patients with incomplete data or follow-up, who missed clinical and laboratory follow-up, and those who refused to participate in the study and failed to sign the Consent Form. These excluded patients were not considered for evaluation. Data collection was performed by one author and histologic exam by two pathologists, in order to minimize interpretation variability among observers. Pathologic exam was performed in a non-blind manner, that is, the pathologists had access to all clinical and laboratory data. The study was approved by the Ethical Committee of the Universidade do Vale do Itajaí.

Use of intravesical therapy (IVT) was determined previously to inclusion and during follow-up: none IVT, use of intravesical mitomicin C, intravesical immunotherapy with BCG and combined mitomicin C and BCG IVT. Mitomicin C administration was performed in the post-surgical period after up to 12 hours of TURS. BCG was administered using induction protocol (once a week during 6 weeks after 2 to 4 weeks of TURS) and maintenance protocol (one series of three weekly cycles of IVT during 6 weeks repeated at 3, 6, 12, 18, 24, 30 and 36 months after induction protocol). A second bladder TRUS was performed 2 to 6 weeks after the initial TURS and always when it was detected incomplete initial resection or absence of muscle, T1 and/or high grade except primary CIS, according to EAU guidelines (5).

Follow-up included urological consultation, urethrocistoscopy, urinary cytology and image exams. It was performed every 3 months in the first 2 years, every 6 months in the next 3 years, and annually until the end of the study and/or one of the end-points. Recurrence was defined as a new lesion after the treatment of the primary tumor diagnosed by cystoscopy and/or image exam and confirmed by histopathology.

The six parameters described by EORTC study (4) were analyzed: histologic grade, pT stage (TNM 2009), size and number of tumors, presence of in situ carcinoma (CIS) and previous recurrence rate. Risk scores (Table-1) and recurrence probability in 1 and 5 years and time to first recurrence (TFR) were estimated in order to compare them with the data of the study by Silvester et al. (4) and accuracy analysis. Patients were divided in 4 risk groups according to their scores (Table-2) and it was determined the number of patients in risk of TFR in 1 and 5 years.

**Table 1:** Recurrence predictors for risk score calculus.

Factor		Recurrence
**Number of tumors**		
	Single	0
	2 to 7	3
	>or=8	6
**Tumor diameter**		
	<3.0cm	0
	>or=3.0cm	3
**Primary previous recurrence**		
	<or=1 recurrence/year	2
	>1 recurrence/year	4
**Cathegory**		
	pTa	0
	pT1	1
**Concomitant CIS**		
	No	0
	Yes	1
**Histologic grade (OMS 1973)**		
	G1	0
	G2	1
	G3	2
**Total score**		0–17

**Source** - Babjuk M et al., 2013

**Table 2:** Score system for NMIBC risk of recurrence calculus.

Recurrence score	Recurrence probability in 1 year		Recurrence probability in 5 years		Recurrent risk group
	%	**(95% 1A)**	%	**(95% 1A)**	
0	15	(10–19)	31	(24–37)	Low risk
1–4	24	(21–26)	46	(42–49)	Intermediate risk
5–9	38	(35–41)	62	(58–65)	Intermediate risk
10–17	61	(55–67)	78	(73–84)	High risk

Source - Babjuk M et al., 2013

Five end-points were analyzed for the end of the study: free of disease, death, death due to disease, noncompliance with treatment, and disease progression.

### Statistical analysis

SPSS software (version 17, SPSS, Chicago Illinois) was used. For statistical validation, recurrence rates at 1 and 5 years were determined. TFR was estimated by Kaplan-Meier method. Validation method included discrimination by accordance index (C index), representing the probability of accordance between predicted value (EORTC) and observed (10). When C is equal to 0.5, there is no discrimination (random distribution) or when it is 1, there is discrimination with perfect accordance (11). An adequate C index was assumed when it was equal or higher than the one of Sylvester et al. study (4). Accuracy was determined by calibration between recurrence probabilities in 1 and 5 years obtained by the present study and by Silvester et al. (4).

## RESULTS

Table-3 shows patients characteristics and comparison with Siylvester et al. data (4).

**Table 3:** Sample characteristic of the present study and of EORTC series.

Characteriscs		Our population n (%)	EORTC n (%)
Total number of patients		205	2596
**Age (years)**			
	≤60	55 (26.8)	859 (33.1)
	61–70	86 (42)	890 (34.3)
	71–80	55 (26.8)	690 (26.6)
	>80	9 (4.4)	118 (4.5)
	unknown	0	39 (1.5)
**Gender**			
	male	144 (70.2)	2044 (78.7)
	female	61 (29.8)	515 (19.8)
	unknown		37 (1.4)
**T stage**			
	pTa	91 (44.4)	1451 (55.9)
	pT1	114 (55.6)	1108 (42.7)
**Number of tumors**			
	Single	97 (47.3)	1465 (56.4)
	2 to 5	64 (31.2)	836 (32.2)
	>5	44 (21.5)	255 (9.8)
	unknown	-	45 (1.7)
**Tumor size**			
	<3cm	103 (50.2)	2087 (80.4)
	≥3cm	102 (49.8)	464 (17.9)
	unknown	0	45 (1.7)
**CIS presence**			
	Yes	21 (10.2)	113 (4.4)
	No	184 (89.8)	2440 (94.0)
**Recurrence**			
	Primary	137 (66.8)	1405 (54.1)
	≤1/year	35 (17.1)	505 (19.5)
	>1/year	33 (16.1)	645 (24.8)
**Histologic grade**			
	Low grade(G1/G2)	95 (46.3)	2260 (87.1)
	High grade (G3)	110 (53.7)	271 (10.4)
**Intravesical therapy**			
	None	90 (43.9)	561 (21.6)
	Chemotherapy	24 (11.7)	2035 (78.4)
	BCG	46 (22.4)	-
	Chemotherapy + BCG	45 (22)	361 (13.9)
**Follow-up (months)**			
	Medium	63.6	46.8
	Minimum	6	-
	Maximum	144	177.6
**Recurrence**			
	Yes	117 (57.1)	1240 (47.8)
	No	88 (42.9)	1356 (52.2)
**End-point**			
	Free of disease	127 (62)	1743 (67.1)
	Progression	25 (12.2)	279 (10.7)
	Death by disease	21 (10.2)	262 (10.1)
	Death	15 (7.3)	461 (17.8)
	Noncompliance with treatment	17 (8.3)	130 (5)
**Survival**			
	Alive	152 (74.2)	1743 (67.1)
	Dead	36 (17.5)	279 (32.9)
	Unknown	17 (8.3)	-

**Figure 1:**
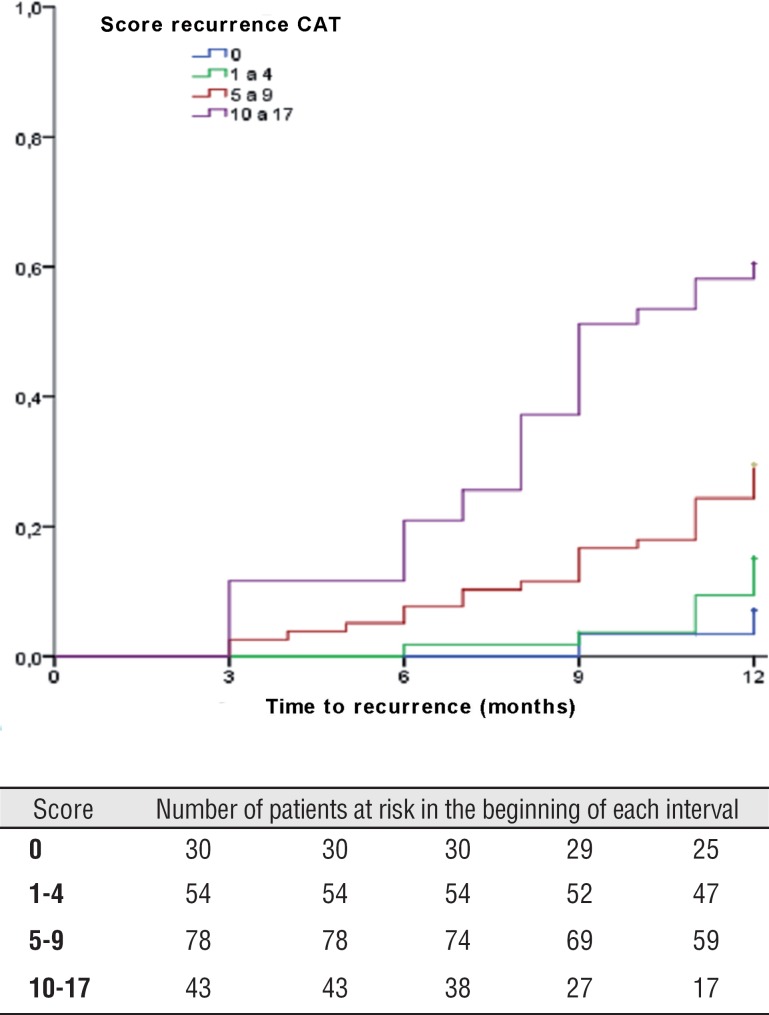
Time to first recurrence in 1 year according to EORTC score in this series.

**Figure 2:**
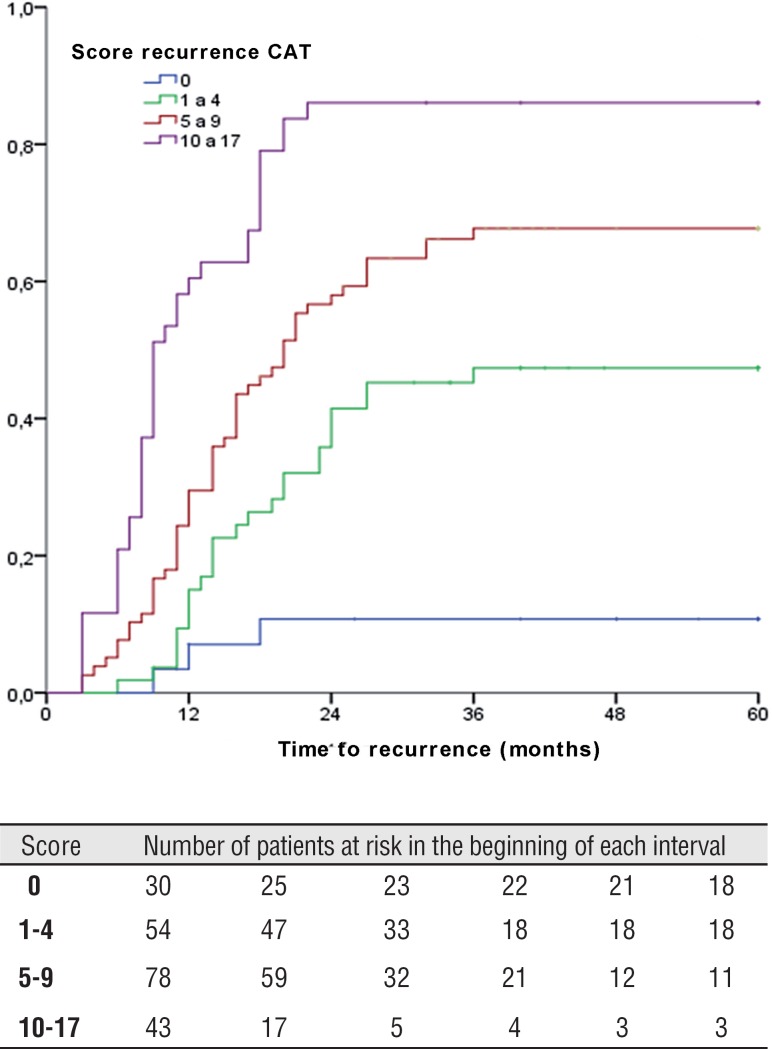
Time to first recurrence in 5 years according to eortc score in this series.

Tumor recurrence was observed in 117 (57.1%) patients. 137 (66.5%) presented primary tumor and 68 (33.2%) previous recurrent tumors, among which 35 (17.1%) with less than 1 recurrence per year and 33 (16.1%) with more than 1 recurrence per year. TFR medium was 14.2±months, minimum 3 and maximum 36 months. Recurrence in 1 year was identified in 59 (28.8%) patients and in 5 years in all 117 (571%) patients. Figures 1 and 2 show TFR in 1 and 5 years, respectively, stratified according to risk score groups of EORTC and show the number of patients at risk in each interval of 3 months, when TFR was evaluated in 1 year, and every 12 months, when it was evaluated in 5 years.

Table shows the number of patients at risk in every interval of 3 months.

Table with the number of patients at risk at intervals of 12 months.

Calculated C index for tumor recurrence was 0.72 for 1 year and 0.7 for 5 years, superior to 0.66 described by Sylvester et al. (4) for the same periods.

Risk of recurrence was 28.8% in 1 year and 57.1% in 5 years, regardless the score. Comparison of probabilities of recurrence in 1 and 5 years with Sylvester et al. results (4) in each group of risk score is shown in Table-4. Probability of recurrence in 1 year was lower in all risk groups than those described by Sylvester et al. (4). In 5 years, risk was superior, except in risk group score zero. For this sample, EORTC Tables overestimated the risk of recurrence in 1 year and underestimated in 5 years, except when score was zero, but the confidence interval overlapped.

**Table 4:** Comparision of recurrence probabilities according to risk score of EORTC and of this study in 1 and 5 years

Recurrence score	number of patients*	Probability of recurrence in 1 year (IC 95%)		Probability of recurrence in 5 years (IC 95%)	
		EORTC	Present study	EORTC	Present study
0	30	15 (10 - 19)	6.7 (0.8 - 22.1)	31 (24 - 37)	10.0 (2.1 - 26.5)
1 - 4	54	24 (21 - 26)	14.8 (6.6 - 27.1)	46 (42 - 49)	46.3 (32.6 - 60.4)
5 - 9	78	38 (35 - 41)	29.5 (19.7 - 40.9)	62 (58 - 65)	66.7 (55.1 - 76.9)
10 - 17	43	61 (55 - 67)	60.5 (44.4 - 75.0)	78 (73 - 84)	86.0 (72.1 - 94.7)
	205		28.8 (22.7 - 35.5)		57.1 (50.0 - 63.9)

* number of patients in the presente study in each risk group of EORTC

## DISCUSSION

BC has a higher incidence in men, 3 to 4 times higher than in women. Median age of patients with BC is 70 years (12). Sylvester et al. (4) identified a relationship of men/women of 3.96 with 80% of male patients and median age of 65 years. We observed a 2.3/1 proportion of men/women with 70.2% of male patients and a median age of 66.61 years. This observed difference may have occurred to the increasing smoking habitus and higher search for preventive medical attention of women observed in daily practice.

Our sample showed higher tumor potential aggressiveness and of high risk than literature (1, 3) and Sylvester et al. study (4) due to a higher proportion of patients stage T1 than literature (1, 3) (5.6% versus 20%) and of high grade tumors (53.7 versus 10.7%), multiple (52.7% versus 42%), larger than 3cm (49.8% versus 17.9%) and with CIS (10.2% versus 4.4%) than Sylverster et al. study (4). Also, the reduced number of CIS cases in Sylvester et al. (4) study lead to low accuracy of EORTC risk tables to predict recurrence and progression of these patients (3). However, the present study observed the same proportion of CIS described in literature (1, 3, 5) expressing a population close to reality.

Pillai et al. (9) described some limitations of Sylvester et al. study (4): collection of data by a single researcher and pathologic exam by a single pathologist may minimize interpretation variability among observers. The present study has not shown significant variability among observers, reducing the possibility of bias of interpretation and analysis of data.

Sylvester et al. (4) based their study in 7 clinical trials, that used several IVT for adjuvant treatment following TURS. In their sample, 78.4% of patients received IVT, a number significantly higher when compared to present study and to the actual need on clinical daily practice. Intravesical chemotherapy protocols used by Sylvester et al. (4) were old and the use of instillation of chemotherapy drug right after surgery, protocol of induction and maintenance of adjuvant intravesical BCG and re-TURS were not considered for the development of EORTC risk Tables. In our sample, 43.9% of patients did not receive any adjuvant IVT. In the other 56.1% patients, 11.7% received chemotherapy using mitomicin C, 22.45 BCG and 22% a combination of Mitomicin C and BCG. The high use of intravesical chemotherapy and the old protocols used by Sylvester t al. (4), the improvement of chemotherapy administration and the increased adjuvant use of BCG nowadays may reduce the predictive power of the EORTC Tables.

Although submitted to adequate treatment, up to 70% of patients with Ta and T1 tumors will present recurrence in 1 year following TURS as single treatment (1, 13). Sylvester et al. (4) reported 47.8% of recurrence in a 14-year follow-up and 44.3% with at least one recurrence when admitted to study. Among these, recurrence occurred at least once a year in 19.5% and more than once a year in 24.8%. Our results are slightly close to those reported in literature (1, 3, 5) than those of Sylvester et al. (4). Recurrence rate was 57.1% in the 12-year follow-up. Also, 33.2% of patients had already presented previous recurrence prior to admittance to study. Among these, 17.1% with less than one recurrence per year and 16.1% with more than one recurrence per year. The lowest recurrence observed by Sylvester et al. (4) when compared to literature (1, 3, 5) and to this study may be explained by the significant difference of patients that received IVT (78.4% versus 56.1%). This fact may have been caused by a selection bias of Sylvester et al. study (4), that included patients from clinical studies proposed to analyze prophylactic value of IVT following TURS and not the study of prognostic factors of recurrence and progression. A vicious sample was selected, since almost 80% of patients received IVT.

In a medium follow-up of 5.5 years with maximum 12 years, among 117 patients that presented recurrence, TFR was 14.2±7.3 months, minimum 3 months, maximum 36 months. Recurrence in 1 year was verified in 28.8% and in 100% in 5 years, while in Sylvester et al. study (4) TFR was 31 months. It was observed more than 50% lower TFR in the studied population. But the time graphic of stratified recurrence of risk groups presented a similar behavior of that of Sylvester et al. (4), mainly at 5 years. In our patients, recurrence was observed in a significant earlier time that could be explained by the lack of use of intravesical chemotherapy and due to tumor characteristics previously described.

Evaluated end-points were similar to those of Sylvester et al. (4) except for death, with a significant difference in this study (32.9% versus 7.3%). Their study showed a higher proportion of men, with a usually lower life expectancy that can explain this difference between studies. Follow-up was similar to Sylvester et al. (4), although lower in total (144 versus 177.6 months) but with a medium higher time (66 versus 46.8 months).

Our C index was 0.72 for recurrence in 1 year and 0.7 for 5 years, superior to 0.66 described by Sylvester et al. (4) for both periods. When C indexes are compared, we conclude that there was discrimination or accordance between them. Also, our C-index showed a better performance than those of Sylvester et al. (4) showing a better accordance of our results to clinical reality. This aspect allows this study to be applied in daily practice.

In relation to accuracy, risk tables overestimated the recurrence risk in 1 year and underestimated in 5 years, probably due to the low use of BCG and high use of intravesical chemotherapy with old protocols utilized by the Sylvester et al. study (4), the use of immediate intravesical chemotherapy with more modern drugs, more adequate use of BCG and routine use of re-TURS in the high risk groups of our study.

In a recent systematic review, Kluth et al. (14) pointed out the importance of treatment impact on end-points of the predictive models and the need of external validations before incorporation in daily practice. External validations of EORTC tables are shown in Table-5.

**Table 5:** Comparision of external validation studies of risk tables of EORTC.

	Number of patients	Sample studied	Studied variables	Endpoints	Accuracy calibration	C-index	External validation
**Fernandez-Gomez et al. (** **8** **)**	1062	Spain	Number of tumors, size, previous recurrence rate, T stage, tumor grade, concurrent CIS	RR 1 and 5 years stratified according to risk groups	Overestimated RR	0,63 for 1 and 5 anos	YES
**Pillai et al. (** **9** **)**	109	United Kingdom	=	RR 1 and 5 years stratified according to risk groups	Underestimated RR in all risk groups	0,62 for 1 year 0,63 for 5 years	NO
**Xylinas et al. (** **23** **)**	4689	Spain	=	RR in 57 months	Overestimated RR mainly in high risk patients	0.597	NO
**Ding et al. (** **15** **)**	301	China	-	RP in 1 and 5 years	Overlapped CI	NR	YES
**Sakano et al. (** **24** **)**	592	Japan	-	recurrence-free survival	NR	NR	NO
**Borkowska et al. (** **25** **)**	91	Poland	=	RR in 1 year stratified according to risk groups	Overestimated RR	NR	NO
**Xu et al. (** **17** **)**	389	Taiwan	-	RR 1 and 5 years stratified according to risk groups	Overestimated RR	NR	YES
**Seo et al. (** **18** **)**	251	South Korea	=	RR 1 and 5 years stratified according to risk groups	Overestimated RR	NR	YES
**Ather and Zaidi (** **20** **)**	92	Pakistan	=	RR in 1 year	Underestimated RR	NR	YES
**Altieri et al. (** **21** **)**	259	Italy	=	RR in 1, 3 qnd 5 years	Overestimated RR in high and intermediate risk groups	NR	YES
**Ajili et al. (** **19** **)**	112	Tunis	=	RR in 1 year stratified according to risk groups	Overestimated RR except in high risk patients	NR	YES
**Hernández et al. (** **16** **)**	417	Spain	=	RR 1 and 5 years stratified according to risk groups	Overlapped CI	NR	YES
**Lammers et al. (** **26** **)**	728	Holand	-	RP in 1 and 5 years	Overlapped ci	NR	YES
**van Rijin et al. (** **22** **)**	230	Multicenter	=	RR 1 and 5 years stratified according to risk groups	-	NR	YES
**Almeida et al.**	205	Brazil	=	RR 1 and 5 years stratified according to risk groups		0,72 for 1 year 0,7 for 5 years	YES

**RR** = Recurrence rate; **RRI** = recurrence risk; **RP** = Recurrence Probability; **IC** = confidence interval; **NR** = no reported = number of tumors, size of tumors, previous recurrence rate, T stage, tumor grade, concomitant CIS

Fernandez-Gomez et al. (8) performed external validation of EORTC risk Tables in patients treated with intravesical BCG, although overestimated the recurrence risk. Ding et al. (15) and Hernandez et al. (16) validated the EORTC model, since the confidence intervals of recurrence rates matched those of Sylvester et al. (4). Xu et al. (17) also validated the model in patients treated with intravesical epirrubicin, although the score overestimated the recurrence rate. Seo et al. (18) validated the risk Tables, since identified a recurrence rate similar to Sylvester et al. (4). Ajili et al. (19) and Ather and Zaidi (20) identified significant correlation for recurrence in 1 year with the Sylvester et al. study (4). Altieri et al. (21) identified a recurrence rate similar to those of EORTC Tables and confirmed their use essential to daily practice. Van Rijin et al. (22) validated the EORTC Tables with a multicenter study treating patients with primary NMIBC and they also advocated their use in that population.

Pillai et al. (9) showed a significant difference of recurrence probabilities between their study and that of Sylvester et al. (4). An inadequate number of patients did not allow a conclusion for external validation. Xylinas et al. (23) analyzed the discrimination of the EORTC Tables and the CUETO score system and identified that both models overestimated the recurrence risk of patients with high risk, but their study was retrospective and multicenter. Sakano et al. (24) affirmed that the EORTC model could not be used in Japanese patients. Borkowska et al. (25) pointed out that risk scores of EORTC overestimated the recurrence risk but, similar to Sylvester et al. (4) study, their sample did not use modern intravesical protocols, what could have limited their results.

Our study presents several limitations. Although our population was homogeneous and close to clinical reality, the size was significantly smaller than the original EORTC study (4). However, the inclusion of 205 patients was satisfactory after comparison with already published validations; the design study and long follow-up allow us to trust in our results. However, although we had a rigorous control of data inclusion and follow-up, sometimes clinical follow-up is difficult in Brazil. It is common difficulties to re-TURS following 3 months, as recommended, delayed control cystoscopies, delayed ambulatory consultation, and lack of comprehension of the actual severity of the disease by the patients.

The greatest difficulty we observed for external validation was the significant disparity between samples of our study and that of EORTC. Sylvester et al. study (4) was not proposed to create prognostic Tables, as previously described, while our study was proposed to analyze those Tables in a more real population. The great merit of Sylvester et al. study (4) was the development of the idea to stablish a mechanism to calculate the recurrence risk of patients with NMIBC. However, EORTC Tables must be adapted in order to become more reliable and used in daily urological practice.

## CONCLUSIONS

The already accepted use of the EORCT model requires some reflections. There is a need to improve risk tables of recurrence in order to minimize distinct ethnic, geographic and clinical practice differences around the World. Tumor markers and genetic mapping are necessary to identify more precisely the biologic behavior of those tumors.

Although EORTC risk Tables overestimated the recurrence risk of tumor in 1 year and underestimated in 5 years, their external validation in patients with NMIBC in the south region of Brazil was adequate and their use to predict recurrence must be reinforced.

## Abbreviations

BC: = bladder cancer
CIS: = carcinoma in situ
EORTC: = European Organization for Research and Treatment of Cancer
EAU: = European Association of Urology
IVT: = intravesical treatment
NMIBC: = Non-muscle invasive bladder cancer
TURS: = Transurethral resection
TFR: = Time to first recurrence
CUETO: = Club Urológico Español de Tratamiento Oncológico
